# Effects of Cannabidiol on Parkinson's Disease in a Transgenic Mouse Model by Gut-Brain Metabolic Analysis

**DOI:** 10.1155/2022/1525113

**Published:** 2022-03-22

**Authors:** Jinchuan Zhao, Xin Gao, Liangyou Zhao, Yiqing Wang, Jingbo Zhang, Shumin Liu

**Affiliations:** ^1^Heilongjiang University of Chinese Medicine, Harbin, China; ^2^Drug Safety Evaluation Center, Heilongjiang University of Chinese Medicine, Harbin, China; ^3^Institute of Chinese Medicine, Heilongjiang University of Chinese Medicine, Harbin, China

## Abstract

Parkinson's disease (PD) is a common neurodegenerative disease characterized by a disorder of the dopaminergic system in the midbrain, causing classical PD motor symptoms. The therapeutic effect of cannabidiol (CBD) on PD has been a research frontier in recent years. However, the pathogenesis of PD and the therapeutic mechanism of cannabinoid remain unclear. To further study the causes of PD and the effect of CBD on PD, we exposed the PD transgenic mouse model to CBD and then estimated the motorial and postural coordination through a modified swim test. Afterwards, the mechanism was investigated via the histopathology of substantia nigra and the gut-brain metabolic analysis in the approach of UHPLC-TOF-MS. The results showed that CBD significantly improved motor deficits of PD model and protected the substantia nigra area. The metabolic function of fatty acid biosynthesis, arginine biosynthesis/metabolism, butanoate (ketone body) metabolism, *β*-alanine metabolism, and pantothenate/CoA biosynthesis was highlighted in the pathological and therapeutic process along the gut-brain axis. In conclusion, CBD could attenuate PD via the neuroprotective effect on the midbrain. The attenuation of the central nervous system in turn improved motor performance of PD, which might be partially induced by the metabolic interaction between the gut-brain. In view of gut-brain metabolomics, the mechanism of PD pathogenesis and the effect of CBD on PD are highly related to the biosynthesis and metabolism of energy and essential substance.

## 1. Introduction

Parkinson's disease (PD) is a common neurodegenerative disease that affects over three million people worldwide and is characterized by a disorder of the dopaminergic system in the midbrain, causing motor deficits like tremors, muscle rigidity, bradykinesia, and impaired gait [1]. The aggregation of *α*-synuclein (*α*Syn) is a stepwise process leading to oligomeric species and intransient fibrils, which accumulate in neurons called Lewy bodies; this is considered to be the pathogenicity of a family of diseases called synucleinopathy and is the focus of the research on PD molecular pathogenesis in recent decades [2–6]. Latest studies have suggested that the *α*Syn misfolding might begin in the gut and ultimately spread to the midbrain, resulting in *α*Syn-mediated motor deficits and brain pathology dominated by the gut-brain axis [7–9]. However, the mechanisms of gut-brain interaction and pathological pathways involved in PD are still unclear. Although various biomarkers derived from clinical, neuroimaging, genetic and biochemical studies have been proposed, the sensitive, specific, and reliable biomarkers for PD remain elusive [10].

On the other hand, pharmacological treatments that improve PD without causing severe adverse function are unavailable. The pharmacological treatment of PD by western medicine is mainly concentrating on symptom management [11]. Dopamine modulators are first-line therapy in PD, while administrations can carry severe side effects and often lose effectiveness [12]. Thus, there is an urgent demand for safe and effective therapy on PD that targets other systems. The endocrine, immune, nervous, and stress systems that profoundly impact human functioning are tightly linked with each other, whereas there are still numerous uncommonly known facts regarding the communication of the systems [13]. In the perspective of integrative medicine, the enteric nervous system (ENS) is a nervous system of the gut that possesses activities independent from other parts of the autonomic nervous system (ANS), regarded as the third division of ANS alongside the sympathetic and parasympathetic divisions [14]. That could be one of the physiological bases that keep signal communication between gut and brain directly and applied on the PD treatment as an alternative. In addition, neuroprotective and antistress strategies applicable to PD can target nonpharmacological systems like nutrition, exercise, and control of the mental environment [15]. Therefore, an integrative therapy on multiple systems involving multiple systems may contribute to a better solution for PD treatment in the future.

There have been some complementary options reported for the PD pharmacological treatment [11, 16]. Currently, cannabidiol (CBD) is one of the main interesting options for PD because of the identification of its multiple potential targets of action in the central nervous system (CNS) and its therapeutic properties for a range of neurodegenerative diseases [17–19]. CBD, a nonpsychotomimetic phytocannabinoid derived from the *Cannabis sativa*, was found to widely suppress inflammatory signaling and oxidative stress. It has antiparkinsonian potential, as it can alleviate the progression of nigrostriatal damage and protect dopaminergic neurons [20–22]. No severe side effects of controlled CBD intake have been reported yet, even for chronic use and high doses up to 1500 mg/day in humans. The treatment of 60 mg/kg CBD i.p. three times per week for 12 weeks on mice had no significant side effects. Some studies reported that CBD could inhibit hepatic drug metabolism, alter *in vitro* cell viability, and decrease fertilization capacity, which all remain further clarified [23]. In addition, its relaxing effect could help attenuate symptoms emotionally and mentally, in a typical way of integrative medicine, meeting the exceptional demands and growing public enthusiasm for botanical medicines [24, 25]. Recently, the neuroprotective effects of CBD have been reported in animal models of PD [26, 27] that are either cannabinoid-receptor independent by reducing glutamate toxicity mediated by NMDA, AMPA, or kainate receptors or indirectly act on the cannabinoid system by blocking anandamide uptake and inhibiting its enzymatic hydrolysis [28–30]. However, the clinical relevance of CBD based therapies on PD motor symptoms has not been systematically evaluated [31]. The pharmaceutical metabolic pathways and function mode of CBD in the treatment of PD remain further clarified as well.

In the current project, we carried out a modified swimming test to study the impact of CBD on the model's motorial coordination, histopathologic observation of mouse substantia nigra to estimate the pathological change of the model's CNS induced by CBD, and analysis of cerebral and fecal metabolic based on bioinformatics. The aim is to evaluate the therapeutic effect of CBD on PD, illustrate the potential mechanisms of metabolic variation involved in PD etiology or pathology, and explore a possible novel way of treating PD through the gut-brain axis.

## 2. Materials and Methods

### 2.1. Reagents and Equipment

CBD (purity >99.5%, tetrahydrocannabinol undetected) was purchased from Hansu Biotechnology Co., Ltd. (Yunnan, China). Tween-80 was purchased from Suobaolai Biotechnology Co., Ltd., (Beijing, China). Acetonitrile, ammonium acetate, and ammonium hydroxide were obtained from Merck Co. (Darmstadt, Germany). The Triple TOF 6600 Mass spectrometer (AB SCIEX, USA), the 1290 Infinity Ultra high-pressure liquid chromatograph (UHPLC, Agilent, Germany), the 5430R low temperature high speed centrifuge (Eppendorf, Germany), the ACQUITY UPLC BEH Amide column (1.7 *μ*m, 2.1 × 100 mm, Waters, USA) were used.

### 2.2. Animals and Administration

The C57BL/6 and *αSyn* A53T transgenic mice (SPF, from Jackson Lab, USA) of six weeks old were provided by JiCuiYaoKan Biotechnology Co., Ltd. (Jiangsu, China). The mice were housed in a standard condition (room temperature (23 ± 1)°C, relative humidity (60 ± 5)%) for one week before the experiments, and free access to food (standard diet) and filtered water. The C57BL/6 mice were set as the control group (group C, *n* = 8); *αSyn* transgenic mice were randomly divided into a model group (group M, *n* = 8) and CBD administration group (group D, *n* = 8). CBD was dissolved in saline mixed with Tween-80 (16 : 1, v/v), and group D was exposed to the CBD solution i.p. in a dose of 4.3 mg/kg/day (0.1 mL/10 g) for three weeks. Meanwhile, the group C and M were administrated with an equal volume of the solvent. The ethical approval was in accordance with the legislation on the protection of animals used for experiment purposes (Directive 86/609/EEC), and the experiments were permitted by the Institutional Animal Care Committee.

### 2.3. Continuous Swimming Test (CST)

Each tested mouse was placed in a 20 × 30 × 20 cm container of 10 cm depth water at 24°C to swim 1 min continuously. The grading standard of CST was further specified for the early-stage PD referring to the classic designs [32, 33] (Table 1). The observers were blinded to the tested group being scored, and a round average score from three independent observers was counted as the grade of a candidate. For group D, especially, a gain of at least 10 more scores after the CBD administration was counted as an effective case and selected for the further following procedures.

### 2.4. Sample Preparation

The midbrain of the selected mice for following histopathology and metabolic analysis was eviscerated on ice and stored in formaldehyde and −80°C fridge, respectively. The fecal samples were collected 12 h after the last experimental administration in a sterile environment and stored at −80°C.

### 2.5. Metabolic Analysis

The nontargeted metabolic analysis was established using ultra high pressure liquid chromatography (UHPLC) coupled to the quadrupole time-off-flight mass spectrometer (TOF MS) in a standard procedure as below. The midbrain samples were mixed in cool methanol/acetonitrile/water solution (2 : 2 : 1, v/v) after thawed at 4°C and then treated with 30 min ultrasound, 10 min standing, and 20 min 14000*g* centrifuge at 4°C. The vacuum dried supernatant was dissolved in 100 *μ*L acetonitrile aqueous solution (acetonitrile : water = 1 : 1, v/v). After centrifuging 15 min at 4°C, the supernatant was taken as the sample for the MS analysis.

Each portion of 2 *μ*L sample was separated through the UHPLC HILIC column at 25°C in speed of 0.5 mL/min in a random order. The mobile phase was composed of A (water containing 25 mM ammonium acetate and 25 mM ammonium hydroxide) and B (acetonitrile), and the sample was eluted at 4°C in the gradient procedure as follows: 0–0.5 min, 95% B; 0.5–7 min, B from 95% to 65%; 7-8 min, B from 65% to 40%; 8-9 min, 40% B; 9-9.1 min, B from 40% to 95%; 9.1–12 min, 95% B. The first- and second-order spectrum of the eluted samples were collected using Q-TOF MS. The electrospray ionization source conditions were set as following. gas 1: 60; gas 2: 60; curtain gas: 30; source temperature 600°C; ion spray voltage floating: ±5500 V; TOF MS scan *m/z* range: 60–1000 Da; product ion scan *m/z* range: 25–1000 Da; TOF MS scan accumulation time 0.20 s/spectra; product ion scan accumulation time: 0.05 s/spectra; declustering potential: ±60 V; collision energy: 35 ± 15 eV. The second order spectrum was obtained from information dependent acquisition in mode of high sensitivity; exclude isotopes within 4 Da; candidate ions to monitor per cycle: 10.

The raw data in the form of “Wiff” was transferred to “mzXML” by the ProteoWizard software and identified as metabolites using XCMS online in a range of the standard databases [34, 35] and strictly verified manually. All the results were recognized as above the level 2 [36] (at least two orthogonal pieces of information, including evidence that excluded all other candidates).

After verification of quality control and normalization to total peak intensity, the processed metabolic data were analyzed by *R* package “ropls,” where it was subjected to multivariate data analysis, including Pareto-scaled principal component analysis (PCA) and orthogonal partial least-squares discriminant analysis (OPLS-DA). A sevenfold cross-validation and response permutation tests were used to evaluate the robustness of the model. The variable importance in the projection (VIP) value of each variable in the OPLS-DA model was calculated to indicate its contribution to the classification.

### 2.6. Bioinformatics Analysis

The bioinformatics analysis of the differentially expressed metabolites among groups was exhibited using R language and platform “MetaboAnalyst 5.0,” including correlation analysis, functional enrichment analysis, and pathway analysis.

### 2.7. Statistical Analysis

The average score of each group in CST was expressed as mean and standard deviation (means ± SD). To test the variation of CST performance among each group, independent *t*-tests were applied on the CST scores, and a paired *t*-test was applied on the CST scores of each group before and after the drug administration. The metabolites with the VIP value >1 was further applied to Student's *t*-test at univariate level to measure the significance among paired groups. A *P*-value less than 0.05 was considered as statistically significant.

## 3. Results

### 3.1. Continuous Swim Test (CST)

**Figure 1:**
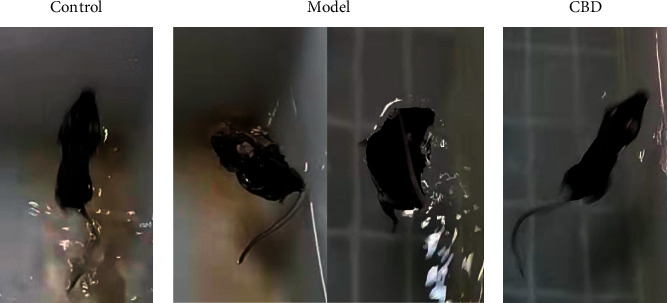
Typical performance of the mice in CST.

The results of CST are presented in Table 2 and Figure 1. The scores of group M and D were significantly lower compared with the group C before administration, demonstrating that the mouse model in early stage of PD has obvious postural and motor incoordination, which is a typical behavioral symptom of PD. Group D gained a significantly higher score by three-week CBD administration than before the administration, indicating that the symptoms of model mice were significantly improved after the administration of CBD.

Particularly, to distinguish the therapeutic effect of CBD on individuals, one in group D gained over 10 scores more than itself before administration was regarded as a significantly effective case (a total of four cases) and was selected for the subsequent further studies.

### 3.2. Histopathology of Substantia Nigra

**Figure 2:**
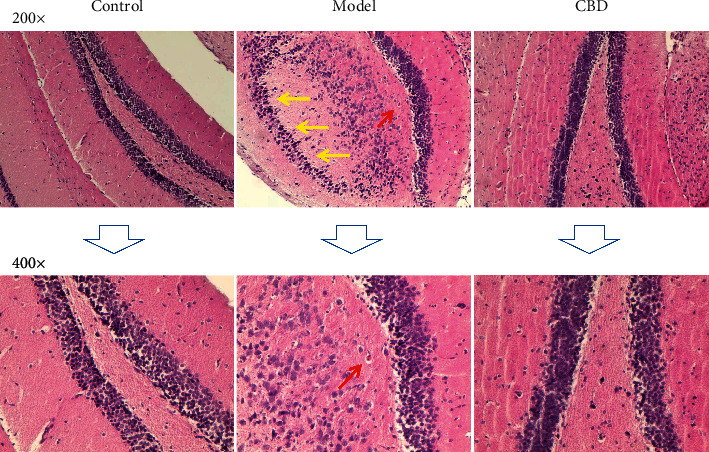
Representative histopathological microphotographs of the substantia nigra area in the midbrain (HE-staining). Yellow arrows indicate obviously fading substantia nigra areas compared twith the control group, and red arrows indicate typical Lewy bodies.

As shown in Figure 2, group M presented degenerative substantia nigra in varying degrees and numerous Lewy bodies around the compacta, which were markers in CNS of PD syndrome. In contrast, group D presented condensed and orderly compacta, similar to group C. No Lewy body was found in groups C and D. It indicated that the administration of CBD on the PD mouse reversed the depletion of substantia nigra and prevented the *α*Syn aggregation visibly.

### 3.3. Gut-Brain Metabolic Functionality and Pathways

**Figure 3:**
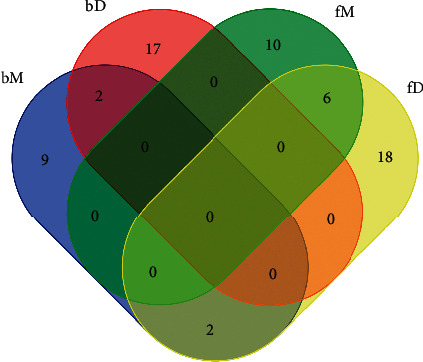
Venn diagram of the four DEMs sets.

By the statistical analysis of paired group comparison and a wide structure-based survey of chemical synonym, four sets of DEMs, namely, bM (brain samples, group M versus C), bD (brain samples, group D versus M), fM (feces samples, group M versus C), and fD (feces samples, group D versus M), were exhibited as in Table 3 in the form of vocabulary executable for bioinformatics analysis. A total of 64 unique DEMs were validated. The mutual DEMs among all the sets were marked to present their variation tendency against those in the corresponding contrast group. The Venn diagram (Figure 3) showed two mutual DEMs between bM and bD, both in the call-back pattern (a reverse trend of DEMs expression between groups); six mutual DEMs between fM and fC, five of which were in the call-back pattern; and two mutual DEMs between bM and fC, one of which was in the call-back pattern.

Based on KEGG pathway mapping, the DEM in the common regulation profile is clustered, focusing on the highly correlated DEMs in a certain set. All DEMs in the call-back pattern were involved in the clustering analysis except ethyl-3-hydroxybutyrate, indicating that the DEMs in a call-back pattern intergroup were highly correlated intragroup as well, which turned into the focused DEMs (Table 3) for the further bioinformatics analysis.

Combined with all available databases on the website MetaboAnalyst 5.0, the functional enrichment and pathway analysis of the four DEMs sets were carried out, respectively.  Table 4 summarizes the significantly enriched terms containing at least two DEMs.

Given the focused DEMs and its corresponding significant functionality or pathway involved, all the results were summarized, and further functional and correlation analysis were proposed referring to the pathway maps and sufficient literature survey.

**Figure 4:**
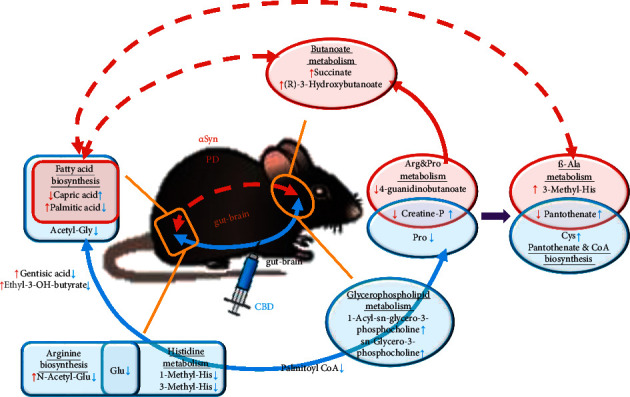
Schematic for focused DEMs and corresponding functionality and pathways. Bold DEMs were highly correlated in a common regulation profile within a certain DEMs set. Red parts stand for PD pathogenesis, and blue parts stand for variation triggered by CBD.

As shown in Figure 4, in the PD mouse model, a significant disorder of butanoate and ketone body metabolism was found in the midbrain characterized by the upregulation of succinate and (R)-3-hydroxybutanoate, which was unique among the four DEMs sets: “arginine and proline metabolism” in a call-back pattern between bM and bD emerged in the same route with the variation of phosphocreatine, 4-guanidinobutanoate and L-proline, pointing at the terminal metabolite 4-aminobutanoate (GABA). As the trigger of ketone body metabolism in cerebral tissue is well known as the utilization of fatty acid, the sole enriched pathway in PD mouse model feces “fatty acid biosynthesis” was also indirectly concluded in the same process through “fatty acid degradation,” involving regulation of capric acid and palmitic acid in a call-back pattern between fM and fD. Considering the focused DEMs and enriched terms in the brain, “arginine and proline metabolism” participated in another pathway branch comprised of 3-methylhistidine (3-MH), pantothenate, and L-cysteine, termed “*β*-alanine metabolism” and “pantothenate and CoA biosynthesis” in a call-back pattern between bM and bD, and this branch was further linked to the disorder of fatty acid biosynthesis in gut intermediated by malonyl-CoA.

Compared with the PD model group, there was downregulation of glutamate and 1-methylhistidine along with call-back of N-Acetyl-L-glutamate and 3-MH in feces of CBD treated group, which was involved in the alteration of arginine biosynthesis and histidine metabolism. It is worth noting that 3-MH and 4-guanidinobutyric acid were the only two metabolites directly linking PD cerebral pathobolism and CBD induced fecal metabolic alteration in the DEMs analysis. In addition, the bD enriched term “glycerophospholipid metabolism” was not merged in the cerebral DEMs network without evident direct or unique connection to others, but linked to the altered process of fatty acid biosynthesis at feces due to a downregulation of palmitoyl CoA and a direct functional connection with palmitic acid.

## 4. Discussion

Since fecal metabolome can provide an avenue to fingerprint the functional status of intestinal microbiota and explore links between the microbiome and host phenotypes, fecal metabolomics has been widely used in biomarker identification and functional annotation for various diseases involved in gut microbiome study [10, 37]. Accumulating evidence suggested that microbiome dysbiosis and changes in microbial metabolite levels are closely related to the pathogenesis of PD [10]. Several neuroactive metabolites were speculated to be regulated by gut microbiota, which in turn affect neuronal activity, such as GABA, tryptophan precursors and metabolites, serotonin, catecholamines, and short chain fatty acids (SCFAs) [38]. These factors can signal to the host via receptors on local cells within the gut and via neurocrine pathways and endocrine mechanisms to targets well beyond the gastrointestinal tract, including vagal afferents in the portal vein and receptors in the brain. One well-studied example is that the ketone bodies can enter the colon and be converted into SCFAs. Different types of SCFA receptors have been identified on enteroendocrine cells and neurons of the submucosal and myenteric ganglia [39].

The present study results verified part of the previous research or speculation. In the PD mouse model, obvious disorder of ketone body metabolism, reduction of phosphocreatine, and pantothenate in midbrain along with fatty acid biosynthesis in feces were detected simultaneously. In normal brain function, threonine and glycine can be converted into creatine, which in turn provides phosphate groups for ADP to produce ATP; with the beginning of *α*Syn aggregation during the onset of the neurodegenerative processes in PD, the metabolism of glycine serine and threonine, as well as the TCA cycle, appear to be downregulated, indicating an energy insufficient and mitochondrial dysfunction in PD [40]. Logically, the energy insufficiency and mitochondrial dysfunction marked by the decrease of phosphocreatine have complementary effects on the increase of ketone body metabolism and degradation of fatty acid, leading to the accumulation of succinate and (R)-3-hydroxybutanoate, both of which act as the terminal metabolites in butanoate metabolism (Figure S1). In turn, the production of ketone body metabolism in brain is related to the variation of fatty acid biosynthesis in gut, marked by the decrease of capric acid and the increase of palmitic acid. Another pathway branch linking the gut “fatty acid biosynthesis” alteration to the brain is the *β*-alanine metabolism marked by the reduction of pantothenate and the increase of 3-MH, which is a marker of Alzheimer's disease in the CSF database (human cerebrospinal fluid). The role of pantothenate in cerebral energy metabolism involved in PD has been figured out [10]; 3-MH is well known as an important functional protein of muscle. It is released when muscle contraction causes the decomposition of contractile protein. Meanwhile, there is no corresponding enzyme for its catabolism and recycling in muscle cells. Thus, the intensity of 3-MH is one of the important indicators of physical motorial status. Histidine can be transported into the brain through the blood-brain barrier and has potential functional significance due to the effect of histamine on the four known *G* protein-coupled histamine receptors, three of which are abundantly expressed in the brain [41]. Therefore, the accumulation of 3-MH in the midbrain may signal the enhancement of somatic 3-MH metabolism, which can be driven and regulated by the histidine metabolism originating from microbiota in the gut. This pathway branch involves cerebral “*β*-alanine metabolism” and fecal “histidine metabolism” and might be a novel research aspect for PD pathological investigation and intervention on the gut-brain axis.

The gentisic acid and ethyl-3-hydroxybutyrate might be other possible novel spots of DEMs involved in PD pathogenesis along the gut-brain axis. Since dopamine is the closest derivative of tyrosine, the depletion ought to be the initial reflection of tyrosine metabolism disorders. Gentisic acid and succinate are the terminal DEMs of the bifurcated pathway in the tyrosine metabolism, and both are upregulated (Figure S2), presumably as a signal of reduction of dopamine generation. Given that gentisic acid is one of the crucial nodes along the path from “tyrosine metabolism” to “butanoate metabolism,” it is suggested to be involved in the initial pathway to “butanoate metabolism” led by maleate. Interestingly, the increase of gentisic acid and ethyl-3-hydroxybutyrate was detected in the gut and not enriched in a certain pathway with the other DEMs, implying that there is still no evidence to verify their role in the pathway along the gut-brain axis for PD pathogenesis. One reasonable explanation for that is that gentisic acid and/or ethyl-3-hydroxybutyrate participate in the SCFAs-like fatty acid biosynthesis process in the gut, which can interact with the cerebral pathology. All the previously mentioned, however, call for further experimental validation.

Since 90% of dopaminergic neurons die in the late stage of PD [42], CBD treatment is likely to be ineffective through inhibiting dopamine recapture in the striatum synaptosomes [43], a model in the early stage of PD was adopted in this study to evaluate the therapeutic effect of CBD on PD. It has previously been proven that cannabis has a beneficial effect on tremor and stiffness, a minor effect on bradykinesia, and a tendency to improve posture, all of which are motor symptoms of PD [44]. To measure motor function, the four most commonly used and verified tests in the PD model are beam traversal, pole descent, nasal adhesive removal, and hindlimb clasping reflexes [45], while the CST test is rarely used. The CST results demonstrated that the performance of mice between control and model groups and before-after CBD administration was well discriminated in this study under a novel modified grading standard with the penalty mechanism. The sensitivity-amplified CST design can provide a complementary method to evaluate PD symptoms comprehensively.

Combining the results of CST, histopathology of substantia nigra, and gut-brain metabolic analysis, we draw a conclusion that three-week i.p. administration of CBD displays an attenuating effect on PD in the early stage, which is reflected in a multifunctional regulation along the gut-brain axis at the metabolic level. The most significant effect of CBD intervention on the PD mouse model was the call-back or regulation of metabolites both in the gut and brain: the upregulated capric acid and downregulated palmitic acid and acetylglycine symbolize a reverse regulatory process of fatty acid biosynthesis in the gut involved in the PD pathogenesis originating from brain metabolic disorders, along with a downregulation of gentisic acid and ethyl-3-hydroxybutyrate. The alteration of arginine biosynthesis and histidine metabolism characterized by downregulating N-acetyl-L-glutamate, L-glutamate, 1-methylhistidine, and 3-methylhistidine was linked to another triggered pathway branch termed “arginine and proline metabolism” that is closely related to the alteration of “pantothenate and CoA biosynthesis” in the midbrain. As metabolome reflects the terminal physiological production logically, the call-back and reverse regulation of symbolic metabolites in a pathological model is a signal or consequence of an effective therapy that has been validated in this study partially.

Interestingly, the concentration variation of (N-acetyl-) L-glutamate occurred in the gut rather than in the brain after CBD administration. The oxidative characteristics of the substantia nigra pars compacta can increase glutamate levels and finally induce the processes of excitotoxicity and neuronal death [46]. Hampson et al. reported that CBD can prevent glutamatergic neurotoxicity and cell death induced by oxidative stress *in vitro*. As CBD is a lipophilic structure, it can cross the blood-brain barrier and has neuromodulation and protection effects on specific areas of the CNS [47]. Additionally, cannabinoids may execute on the reuptake of glutamate in the brain [43]. A statistically significant alteration of glutamate level was identified in blood samples as well from PD [48]. Meanwhile, the feces-related disease obesity was extremely significantly enriched in CBD treated group involving L-glutamate, as the results showed, indicating that the direct function of CBD on the gut microbiota cannot be excluded. Because the concentration of N-acetyl-L-glutamate in tissues is considered as the indicator of cellular glutamate concentration, the decline of both is seen as a whole for call-back regulation of the glutamate level increase in the gut of the PD model. All the current evidence suggests that the gut and the brain could interact in the glutamate metabolism of PD and probably through the blood circuit intermediately, while CBD can reverse the pathological process in a still controversial mechanism that whether variation of the metabolite level in the gut/microbiota is the consequence or the initiation of cerebral metabolic disorder, or in a coordinative way.

Due to the organ variety, it is unsurprising that there is hardly a direct metabolites connection between gut and brain. Upon a primary DEMs analysis in the approach of the regulation profile, we managed to build reasonable metabolic linkages along the gut-brain axis further by the function and pathway analysis. In that way, part of focused metabolites became involved in a meaningful biological or pathological explanation for PD pathogenesis and CBD's therapeutic effect. This study all relies on the currently available prior databases and knowledge; the mechanism of the inferred results requires further experimental validation; and the unmentioned or unanalyzed part might be still worth mining.

## 5. Conclusion

CBD could attenuate PD via the neuroprotective effect on the midbrain. The attenuation of the central nervous system in turn improved motor performance of PD, which might be partially induced by the metabolic interaction between the gut-brain. In view of gut-brain metabolomics, the mechanism of PD pathogenesis and the effect of CBD on PD are highly related to the biosynthesis and metabolism of energy and essential substance.

## Figures and Tables

**Table 1:** Grading standard of CST.

Score	Description
30	Full score for a normal individual swimming continuously in regular posture
25	Swim fluently with once short break (<2 s)
20	Swim fluently with casual short break (<2 s)
15	Swim with frequent break or floating (<2 s)
10	Swim less than half time
0	Swim not at all
−5	Penalty for incoordinative posture, e.g., indolent swim or stiff tail
−10	Penalty for poor posture, e.g., spiraling swim or erect tail
−10	Penalty for long floating (>2 s)

**Table 2:** Grading of CST.

Group	Control (C)	Model (M)	CBD (D)
Before ad.	26.25 ± 1.25	13.13 ± 2.30^Δ^	12.50 ± 1.89^Δ^
After ad.	26.25 ± 1.57	12.50 ± 2.32	17.50 ± 1.89^*∗*^
Improved individuals (proportion)	1 (12.5%)	2 (25%)	4 (50%)

Δ*P* < 0.001 significant different from group C; ^*∗*^*P* < 0.05 significantly different from the same group before the administration (ad.).

**Table 3:** Focused cerebral and fecal DEMs of each paired group comparison.

DEMs set	bM	bD	fM	fD
Call-back pattern intergroup	**↓Pantothenate** **↓Phosphocreatine** **↑3-methylhistidine**	**↑Pantothenate** **↑Phosphocreatine**	**↓Capric acid** **↑Gentisic acid** **↑Palmitic acid** **↑N-acetyl-l-glutamate**↑Ethyl-3-hydroxybutyrate	**↑Capric acid** **↓Gentisic acid** **↓Palmitic acid** **↓N-acetyl-l-glutamate**↓Ethyl-3-hydroxybutyrate**↓3-methylhistidine**
**Highly correlated intragroup**	**↑Succinate** **↑(R)-3-hydroxybutyric acid** **↓1,5-bisphosphate** **↓D-Mannose 1-phosphate** **↓Galactonic acid** **↓4-guanidinobutyric acid**	**↑L-cysteine↓Prostaglandin E2↓Palmitoyl CoA↓cis-Aconitate↓2-dehydro-3-deoxy-D-gluconate**		**↓L-glutamate↓1-ethylhistidine↓Betaine↓Nicotinate↓Anthranilic acid↓5-methylcytosine↓4-guanidinobutyric acid**

↑ upregulated, ↓ downregulated, compared with the corresponding contrast group. bM: brain samples, group M versus C; bD: brain samples, group D versus M; fM: feces samples, group M versus C; fD: feces samples, group D versus M. The DEMs in form of bold within a certain group are highly correlated in a common regulation profile according to the KEGG pathway based clustering analysis.

**Table 4:** Significantly enriched terms in the DEMs bioinformatics analysis and the corresponding involved DEMs.

DEMs set	Analysis (database)	Terms (≥2 DEMs)	Involved metabolites	Sig.
bM	Enrichment/pathway (SMPDB & KEGG)	**Ketone body**/**butanoate** metabolism	**Succinate; (R)-3-hydroxybutyric acid**	^*∗∗*^
		**Beta-alanine** metabolism	**Pantothenate; 3-methylhistidine**	^*∗*^
		**Arginine** and **proline** metabolism	**Phosphocreatine; 4-guanidinobutyric acid**	^*∗*^
bD	Pathway (KEGG)		**Phosphocreatine**; L-proline	^*∗*^
		Glycerophospholipid metabolism	1-Acyl-sn-glycero-3-phosphocholine; sn-Glycero-3-phosphocholine	^*∗*^
	Enrichment/pathway (KEGG/SMPDB)	**Pantothenate** and CoA biosynthesis	**Pantothenate; L-cysteine**	^*∗*^
fM	Enrichment/pathway (KEGG)	**Fatty acid** biosynthesis	**Capric acid; palmitic acid**	^*∗*^
fD	Pathway (SMPDB/KEGG)		**Capric acid; palmitic acid**; acetylglycine	^*∗*^
	Enrichment (human feces)	Obesity	**L-glutamate**; vaccenic acid	^*∗∗∗*^
	Enrichment (SMPDB/KEGG)	**Arginine** biosynthesis	**L-glutamate; N-acetyl-L-glutamate**	^*∗*^
		**Histidine** metabolism	**L-glutamate; 3-methylhistidine 1-methylhistidine**	^*∗*^

bM: brain samples, group M versus C; bD: brain samples, group D versus M; fM: feces samples, group M versus C; fD: feces samples, group D versus M. The focused DEMs and the corresponding significant terms were in form of bold. Significance (Sig.): ^*∗∗∗*^*P* < 0.001; ^*∗∗*^*P* < 0.01; ^*∗*^*P* < 0.05.

## Data Availability

The data used to support the findings of this study are available from the corresponding author upon request.

## Supplementary Materials

Supplementary Materials Figure S1: KEGG metabolic pathway map “butanoate metabolism,” with highlighted involved DEMs and pathway terms. Figure S2: KEGG metabolic pathway map “tyrosine metabolism (mouse),” with highlighted involved DEMs and metabolite or pathway terms.
