# Supplementary material for: Effects of Cannabidiol on Parkinson's Disease in a Transgenic Mouse Model by Gut-Brain Metabolic Analysis
Source: Evid Based Complement Alternat Med. 2022 Mar 22;2022:1525113. doi: 10.1155/2022/1525113 (PMC8964161; doi:10.1155/2022/1525113)
Supplement: Supplementary Materials — Figure S1: KEGG metabolic pathway map “butanoate metabolism,” with highlighted involved DEMs and pathway terms. Figure S2: KEGG metabolic pathway map “tyrosine metabolism (mouse),” with highlighted involved DEMs and metabolite or pathway terms. [file 1525113.f1.zip › 1525113.f1/Figure S2 Tyrosine metabolism (mouse).doc]

**fM**

**bM**

**bM**
